# A reinforcement learning approach to gait training improves retention

**DOI:** 10.3389/fnhum.2015.00459

**Published:** 2015-08-27

**Authors:** Christopher J. Hasson, Julia Manczurowsky, Sheng-Che Yen

**Affiliations:** ^1^Neuromotor Systems Laboratory, Department of Physical Therapy, Movement and Rehabilitation Sciences, Northeastern UniversityBoston, MA, USA; ^2^Laboratory for Locomotion Research, Department of Physical Therapy, Movement and Rehabilitation Sciences, Northeastern UniversityBoston, MA, USA

**Keywords:** reinforcement learning, supervised learning, motor learning, retention, gait, reward, adaptation, rehabilitation

## Abstract

Many gait training programs are based on supervised learning principles: an individual is guided towards a desired gait pattern with directional error feedback. While this results in rapid adaptation, improvements quickly disappear. This study tested the hypothesis that a reinforcement learning approach improves retention and transfer of a new gait pattern. The results of a pilot study and larger experiment are presented. Healthy subjects were randomly assigned to either a supervised group, who received explicit instructions and directional error feedback while they learned a new gait pattern on a treadmill, or a reinforcement group, who was only shown whether they were close to or far from the desired gait. Subjects practiced for 10 min, followed by immediate and overnight retention and over-ground transfer tests. The pilot study showed that subjects could learn a new gait pattern under a reinforcement learning paradigm. The larger experiment, which had twice as many subjects (16 in each group) showed that the reinforcement group had better overnight retention than the supervised group (a 32% vs. 120% error increase, respectively), but there were no differences for over-ground transfer. These results suggest that encouraging participants to find rewarding actions through self-guided exploration is beneficial for retention.

## Introduction

Restoration of a healthy walking pattern is a major goal of neurological rehabilitation. To facilitate this process, visual cues and manual or robotic assistance can be provided, which provides patients with feedback about their performance. A critical outcome of such training is how long an improved gait pattern persists after augmented feedback and external assistance are removed, i.e., is there retention? A gait training program is considered to be more effective if patients can retain the learning effect longer after each training session.

Motor learning paradigms can be placed along a continuum based on the guidance provided by a human or machine. Many current gait training approaches fall heavily on one side of this continuum, under the umbrella of supervised learning. In such approaches, either a human or machine acts as a supervisor that guides a patient into a desired gait pattern. Feedback provided typically includes information about error magnitude and direction (i.e., vector-based), and can be provided via visual or haptic (e.g., guidance forces) sensory modalities.

A concern of supervised learning approaches is that the guidance often elicits fast adaption, but poor retention, i.e., patients quickly revert to their old gait patterns when the supervisor is removed. For example, in patients with Parkinson’s disease, Morris et al. (1996) showed relatively little next-day retention of increases in stride length achieved through visual cues. In patients with incomplete spinal cord injury, Yen et al. (2012) found that increases in stride length induced by robotic assistance quickly disappeared after assistance was removed. The asymmetric gait seen in chronic stroke can be addressed with split-belt treadmill training (Reisman et al., 2007, 2009; Malone and Bastian, 2014); subjects can be guided towards or away from a desired level of symmetry through differential modulation of treadmill belt speeds beneath each leg. However, in these split-belt studies, beneficial adaptations rapidly diminish after a few steps.

Understanding why supervision-based gait training approaches have poor retention, and testing alternative approaches, may improve gait rehabilitation outcomes. One explanation may be that supervision-based approaches disengage individuals from the learning process. For example, when the Lokomat® rehabilitation robot is used to assist patients’ stepping during treadmill training, patients tend to reduce their active participation (Israel et al., 2006). Another explanation may be that supervised learning approaches, which provide learners with vector-based (directional) error information, may be more internal model-driven, and could generate competition between a newly learned gait pattern and the old pattern (Shmuelof et al., 2012). Additional insight may be gained by manipulating the way in which a patient attains a desired gait. More specifically, by asking the question: would retention be different if a patient was asked to find the desired gait on their own, instead of being guided?

Self-guided exploration as a means of finding rewarding actions is a hallmark of reinforcement learning (Barto, 1998). Traditional human motor learning studies have generally shown that self-guided practice provides better retention than practice encumbered by heavy supervisory guidance (Lee et al., 1990; Winstein et al., 1994). It could be speculated that finding a new movement pattern on one’s own improves retention because it is a more rewarding experience compared with being led through guidance, as positive reinforcement improves retention (Abe et al., 2011; Pekny et al., 2011; Galea et al., 2015). A reinforcement learning-based gait training approach may also facilitate transfer of gait patterns learned on a treadmill to different contexts (e.g., over-ground), another important gait rehabilitation outcome. The rationale for improved transfer is that increased reward uncertainty may promote information seeking through exploration (Inglis et al., 1997; Anselme, 2010), and more variable practice facilitates generalization (Kerr and Booth, 1978; Wrisberg and Liu, 1991; Green et al., 1995; Sherwood, 1996).

The purpose of this study was to evaluate the effectiveness of a reinforcement-based gait training program, and compare learning outcomes with a supervision-based paradigm. Two experiments were performed. The first was a pilot experiment (Experiment 1) to determine whether subjects could learn a new gait pattern under a reinforcement learning paradigm, i.e., without having knowledge of the desired gait pattern and only receiving categorical error feedback and rewards. After completing the pilot study, the results were deemed to have sufficient merit to repeat the study with a larger sample size, an improved measurement system, and minor changes to the task (Experiment 2). In both experiments, a supervised group of healthy subjects was provided with directional error feedback while they learned a new gait pattern on a treadmill. A second reinforcement group received non-directional error feedback, which only showed whether subjects were close or far from the desired gait. Both groups received artificial monetary rewards for small errors. Three hypotheses were tested: The reinforcement group will improve their task performance, retain what they learned, and transfer their learned gait pattern to an over-ground walking context (Hypothesis 1). The reinforcement group will have better immediate and overnight retention compared to the supervised group (Hypothesis 2). The reinforcement group will have better immediate and overnight over-ground transfer compared to the supervised group (Hypothesis 3).

## Methods

### Experiment 1

#### Experimental Design

Subjects participated in two data collection sessions on two consecutive days. On the first day they practiced a new gait pattern on a treadmill set at a relatively slow speed of 1 mph, as patients with walking disability tend to walk slower. After practicing on a treadmill, immediate (same day) and overnight retention and transfer tests were performed. The transfer tests were performed over-ground, and subjects were asked to walk at a speed similar to that experienced during treadmill adaptation.

#### Subjects

A total of 16 healthy young adults were recruited. They were randomly assigned to either a supervision group (2 males/5 females; age: 22.9 ± 1.6 years; height: 166 ± 7.4 cm; weight: 67.5 ± 14.9 kg), in which the visual feedback was supervisory in nature (i.e., directional error information guiding towards a desired gait goal), or a reinforcement group (3 males/4 females; age: 22.8 ± 0.7 years; height: 170.5 ± 9.3 cm; weight: 70.1 ± 11.2 kg), in which the visual feedback consisted of non-directional error feedback (i.e., categorically close to or far away from a predetermined goal). All subjects were healthy and had no neurological or musculoskeletal issues that affected their balance or ability to walk. The study was approved by the Northeastern University Institutional Review Board, and all subjects signed an informed consent form prior to participation.

#### Task

The goal of the gait training was to have subjects learn a new gait pattern, such that their right ankle reached 10° of eversion, relative to their ankle angle in quiet standing, 200 ms after toe-off during the swing phased of the gait cycle. This desired ankle position was chosen because it represented an unfamiliar gait pattern.

#### Apparatus

The right ankle angle was measured with an electrogoniometer (Biometrics LTD, Ladysmith, VA, USA). The electrogoniometer was positioned over the dorsal surface of the midtarsal joint following the procedure described in Palmer et al. (1998). The superior portion of the electrogoniometer was taped along the anterior surface of the tibia and the inferior portion was taped to the dorsal surface of the subject’s shoe, in line with the second metatarsal. A FSR foot switch (Interlink Electronics, Westlake Village, CA, USA) was placed under the first metatarsal head for detection of toe-off. Voltages representing the electrogoniometer inversion/eversion axis of rotation were sampled with an analog-to-digital converter (Delsys, Natick, MA, USA).

#### Visual Feedback

**Figure 1:**
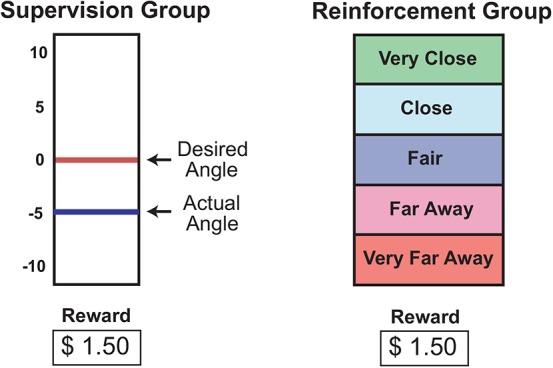
**Visual feedback provided to the supervision and reinforcement groups**.

A custom program written in LabView (National Instruments, Austin, TX, USA) provided error feedback based on the sampled electrogoniometer data. The feedback was shown on a monitor placed in front of the treadmill. The supervised group received directional error feedback (Figure 1). A graduated vertical scale was provided that displayed both the desired ankle position and subject’s actual ankle position 200 ms after toe-off. This display provided information about how the ankle’s angular position should be changed to achieve the desired angle. The reinforcement group received a categorical indication of their ankle position error (Figure 1). One of the following descriptors was provided: “Very Close” (absolute error = 0°–2°), “Close” (3°–5°), “Fair” (6°–8°), “Far Away” (9°–11°), and “Very Far Away (>12°). Note that this feedback does not provide directional information, e.g., “Far Away” does not indicate how a subject should change his/her movement pattern to reduce the error magnitude to “Fair”. For both supervised and reinforcement groups the display was updated after each gait cycle depending on the latest ankle angle error. For additional motivation a fictitious monetary reward was displayed on the monitor for both groups during practice with visual feedback. This amount was proportional to the inverse of the error (small error = high reward), and was cumulative.

#### Instructions

The supervision group was given a physical demonstration of the desired gait pattern and explicit instructions about the task goal. They were told that a positive on-screen error meant that their ankle was too far inwards (inverted), and vice-versa for a negative error (too much eversion). The reinforcement group, on the other hand, was not given a detailed explanation of the task goal. There were told that they were to learn a new gait pattern, and that this new gait involved the movement of the shank and foot segments. They were also told that the “Very Close” feedback indicated that they were very close to the desired gait pattern and that they were progressively farther away as they moved from “Very Close” to “Very Far Away”.

#### Protocol

**Figure 2:**
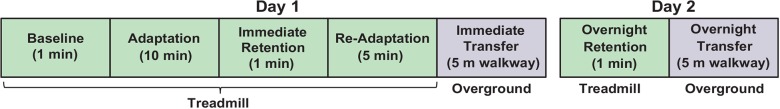
**Experimental protocol**. After practicing the gait task, retention and transfer were tested immediately and after a night of rest.

During the first day of data collection, subjects walked with their nominal gait on the treadmill for 1 min (*baseline*; Figure 2). Subjects then performed the learning task for 10 min (*adaptation*). Next, visual feedback was removed and subjects were told to replicate the desired gait while walking for 1 min *(immediate retention*). After this, visual feedback was displayed again and subjects performed the learning task for 5 min (*re-adaptation*). Subjects then moved to the ground and were asked to replicate the desired gait when they walked over-ground on a 5 meter walkway two times (*immediate transfer*). During the second day, subjects were again asked to replicate the desired gait on a treadmill walking for 1 min without feedback (*overnight retention*) and then in over-ground walking (*overnight transfer*).

#### Data Reduction

The dependent variable was the absolute angular error (difference) between the goal and actual ankle angle 200 ms after toe-off, calculated once per gait cycle. An inspection of the data showed a relatively high amount of within- and between-subject variability, which is likely due to the relatively small sample size. Therefore, only a descriptive analysis was performed for Experiment 1.

### Experiment 2

#### Subjects

A total of 32 healthy young adults were recruited. They were randomly assigned to either a supervision (7 males/9 females; age = 22 ± 2.1 years; height = 168.6 ± 8.9 cm; weight = 64.2 ± 13.2 kg) or reinforcement group (4 males/12 females; age = 23.7 ± 3.3 years; height = 167.9 ± 10.9 cm; weight = 60.1 ± 10.8 kg). There were no significant between-group differences in age (independent *t*-test, *p* = 0.09), weight (independent *t*-test, *p* = 0.40), height (independent *t*-test, *p* = 0.82), and gender distribution (Chi-square test, *p* = 0.3). All subjects were healthy and had no neurological or musculoskeletal issues that affected their balance or ability to walk. The study was approved by the Northeastern University Institutional Review Board, and all subjects signed an informed consent form prior to participation.

#### Task

As in Experiment 1, subjects were asked to learn a new gait pattern. However, the desired amount of eversion was set to 5° instead of 10°. This was because in this experiment (Experiment 2) a motion capture system was used to measure the true eversion angle (see “Apparatus” Section). The electrogonimeter eversion angle measurements used in Experiment 1 were more susceptible to cross-talk from out-of-plane motions, i.e., the electrogonimeter voltages associated with “eversion” could be changed not only by eversion, but to a lesser degree by ab/adduction of the foot. Thus, a greater apparent eversion angle could be achieved with the electrogonimeter by everting and abducting the foot, inflating the range of angles that subjects could achieve. On the other hand, the motion capture-based system measured the true eversion angle, and therefore the range of angles that subjects could achieve was reduced. Consequently, the target angle was reduced to 5°.

#### Apparatus

The relative motions of the shank and foot were tracked using a motion capture system (Qualysis, Gothenburg, Sweden). A cluster of three reflective markers was attached to the shank over the tibia, and another cluster of four reflective markers was attached to the foot. A minimum of three non-collinear markers is needed to determine the three-dimensional orientation of a segment; an extra marker was used for the foot due to instances of marker occlusion. The clusters were attached to a rigid backing plate, which was taped to the body segments using medical tape. Rigid-body models were defined in the Qualysis Track Manager (QTM), and the segment angles were calculated using the standard QTM Euler angle definition. As in Experiment 1, a foot switch was placed under the first metatarsal head for detection of toe-off.

#### Visual Feedback

The visual feedback was the same as in Experiment 1.

#### Instructions

The instructions were the same as in Experiment 1.

#### Protocol

The protocol was the same as in Experiment 1.

#### Data Reduction

The dependent variable was the same as in Experiment 1, i.e., the absolute angular error between the actual and target ankle angle 200 ms after toe-off.

#### Data Analysis

An analysis of the distribution of absolute errors showed a pronounced positive skew; therefore, for statistical analysis a transformation was performed to make the distribution of errors normal. To determine the correct transformation, the *boxcox* MATLAB (Mathworks, Natick, MA, USA) function was used. This function finds the optimum transformation parameter λ that maximizes the log-likelihood function. The optimum λ was equal to zero, and therefore the data were transformed as *x*(*λ*) = log(*x*), where *x* is the absolute angular error.

To test the three hypotheses there were four points of interest: early practice, late practice, immediate retention/transfer, and overnight retention/transfer. For early and late practice, the absolute error was computed for the first 10 and last 20 stride cycles, respectively. Fewer cycles were used in the early practice measures because error changed more rapidly compared to late practice. For immediate and overnight retention tests, the average error was computed across all stride cycles. For over-ground transfer, the average error was taken across the first 5 strides within each trial, and these results averaged between the two trials.

#### Statistical Analysis

To test Hypothesis 1, a linear mixed model analysis for repeated measures was performed to determine whether the reinforcement group learned the task, was able to retain their skill without feedback, and was able to transfer their skill to an over-ground walking context. An unstructured variance model was used, which allowed the observed data to dictate the correlations between repeated time points. A separate linear mixed model analysis was performed for the supervised group, who served as controls. The dependent variable was the log-transformed absolute error, and the independent variable was time.

Significant main effects were followed up by *post hoc* tests to determine if there were significant differences between the following time points of interest: (a) early and late adaptation periods; (b) late adaptation and immediate retention; (c) late adaptation and immediate transfer; (d) immediate to overnight retention; (e) immediate to overnight transfer. Outliers were identified as those subjects that had an average error or error standard deviation larger than *q*_3_ + *w*(*q*_3_ − *q*_1_) or smaller than *q*_1_ − *w*(*q*_3_ − *q*_1_), where *w* = 1.5 and *q*_1_ and *q*_3_ are the 25th and 75th percentiles, respectively. One subject from the supervised group and two subjects from the reinforcement group fulfilled these criteria, and therefore were excluded from the test of Hypothesis 1.

To test Hypotheses 2 and 3, which focused on group differences in retention and transfer, respectively, a between-subjects analysis was performed. To test Hypothesis 2, an independent samples *t*-test was used to compare the change in the log-transformed absolute error from late-practice to immediate retention between the supervised and reinforcement groups. A second *t*-test was performed to test for group differences in the change in error from immediate to overnight retention. To test Hypothesis 3, two more independent samples *t*-tests were performed comparing the relative amount of transfer between late practice and immediate transfer, and between immediate and overnight transfer. One subject was excluded from the reinforcement group using the same outlier criteria as used for testing Hypothesis 1. All statistical analyses were conducted using SPSS (Version 21, IBM Corp., Armonk, NY, USA) with critical *α* = 0.05.

## Results

### Experiment 1

#### Descriptive Analysis

**Figure 3:**
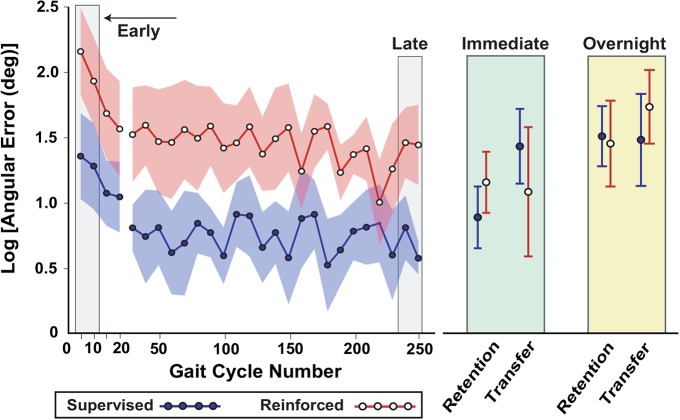
**Descriptive results of pilot study (Experiment 1), showing that subjects could learn a new gait pattern under a reinforcement learning paradigm**. Performance was characterized by the logarithm of the absolute angular error is with respect to the goal ankle position over practice and on retention and transfer tests. Due to the small sample size and high within- and between-subject variability, only a descriptive analysis of this data was performed. At the start of practice the averages are shown for 5-trial bins, while the rest of practice (through late practice) shows 10-trial bins. Shading and error bars indicate the standard error.

Changes in the absolute angular inversion/eversion error across the initial practice period, and on the retention and transfer tests, are shown in Figure 3. During initial practice the reinforcement group appeared to have greater errors compared to the supervised group. Both groups improved their task performance with practice, retained their skill in the immediate retention test, and were able to transfer the learned gait pattern to an over-ground walking context in the immediate transfer test. Overnight, both retention and transfer became worse, but it appeared that the supervision group may have lost more of their skill compared to the reinforcement group.

### Experiment 2

#### Descriptive Analysis

**Figure 4:**
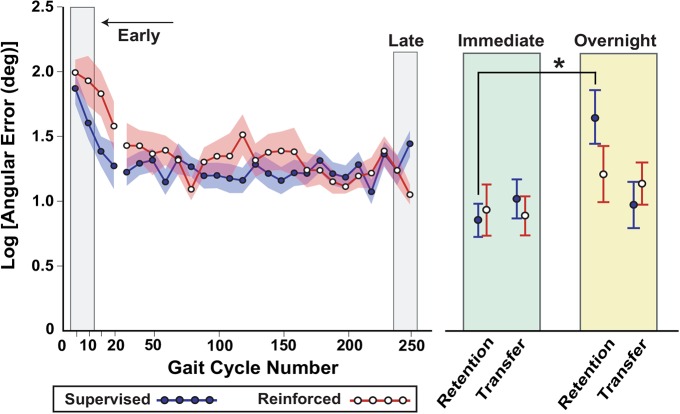
**Results of Experiment 2, showing that the reinforcement group had better overnight retention compared to the supervised group**. As in Experiment 1, performance was characterized by the logarithm of the absolute angular error is with respect to the goal ankle position over practice and on retention and transfer tests. In this experiment the sample size was doubled, an improved measurement system was used, and minor changes to the task were made (the target was reduced from 10° to 5°). At the start of practice the averages are shown for 5-trial bins, while the rest of practice (through late practice) shows 10-trial bins. Shading and error bars indicate the standard error. *Supervised group had a greater increase in error from immediate to overnight retention compared to the reinforcement group (Bonferroni-adjusted *p* = 0.04).

The absolute angular error and performance on the retention and transfer tests is shown in Figure 4. In contrast to Experiment 1, both reinforcement and supervision groups achieved comparable skill levels during practice of the gait task. While immediate retention and transfer were similar between the groups, significant differences in retention emerged overnight. The results of the statistical tests for each of the three hypotheses are presented next.

#### Can Subjects Learn, Retain, and Transfer a New Gait with a Reinforcement Learning Approach (Hypothesis 1)?

In the reinforcement group, the linear mixed model detected a significant time effect on the log transformed error (*F*_(5,13)_ = 5.01, *p* < 0.01). *Post hoc* tests indicated that the error decreased from early to late practice (*t*_(13)_ = 4.3, Bonferroni-adjusted *p* < 0.01, effect size *d* = 1.81 [large effect; Cohen, 1977]). There were no differences in error between: late adaptation and immediate retention, late adaptation and immediate transfer, immediate and overnight retention, and immediate and overnight transfer (all *p* values > 0.11).

In the supervised learning group, the linear mixed model also detected a significant time effect on the log transformed error (*F*_(5,14)_ = 5.03, *p* < 0.01). *Post hoc* tests indicated that the error decreased from early to late practice (*t*_(14)_ = 3.76, Bonferroni-adjusted *p* < 0.01, effect size *d* = 1.4 [large effect]). Error increased from immediate to overnight retention (*t*_(14)_ = 3.25, Bonferroni-adjusted *p* = 0.03, effect size = 1.5 [large effect]). There were no differences in the error between: late adaptation and immediate retention, late adaptation and immediate transfer, and immediate and overnight transfer (all *p* values >= 0.49).

#### Was there a Group Difference in Retention (Hypothesis 2)?

There was no difference in the change in error from late adaptation to immediate retention between the supervised and reinforcement groups (*p* = 0.67). However, the increase in error from immediate to overnight retention was greater in the supervised group compared to the reinforcement group (a 120% [(5.17–2.35)/2.35] vs. 32% [(3.35–2.54)/2.54] increase in error; *t*_(29)_ = 1.2, Bonferroni-adjusted *p* = 0.04, effect size *d* = 0.43 [small to medium effect]). In other words, the supervised group lost more of their skill than the reinforcement group after a day of rest.

#### Was there a Group Difference in Transfer (Hypothesis 3)?

There was no between-group difference in the change in error between late adaptation and immediate transfer (*p* = 0.21). There was also no between-group difference in the change in error between immediate and overnight transfer (*p* = 0.25).

## Discussion

### Main Findings

A preliminary experiment (Experiment 1) showed the feasibility of training healthy subjects in a new gait pattern using a reinforcement learning-based feedback scheme. This experiment was then repeated with a larger sample size, an improved measurement system, and minor changes to the task (Experiment 2). The results of the second experiment showed that subjects who received categorical error information in a reinforcement learning paradigm improved their task performance, retained what they learned, and transferred their learned gait pattern to an over-ground walking context (supports Hypothesis 1). A significant finding was that the reinforcement group had better overnight retention than a supervised group that received more directionally-specific error information (supports Hypothesis 2). There were no group differences in the amount of transfer (against Hypothesis 3). These results suggest that making participants find rewarding actions through self-guided exploration is beneficial for retention.

### Retention of a Novel Gait Pattern

The results showed that a reinforcement learning-based feedback scheme improved retention of a novel gait pattern. This may be because rewards for good performance come less often and are more uncertain, particularly in early learning. It has been demonstrated that behaviors are retained more when rewards are provided intermittently, i.e., when there is partial reinforcement, in animals (Jenkins and Stanley, 1950; Ferster and Skinner, 1957) and humans (Winstein and Schmidt, 1990). Further, a more uncertain reward may act as a stronger motivational agent. Dopamine release increases with greater reward uncertainty in both monkeys (Fiorillo et al., 2003) and humans (Preuschoff et al., 2006; Linnet et al., 2012), and dopamine has a role in post-trial memory consolidation (i.e., retention) in humans (Matsumoto and Hikosaka, 2009). In the present study, although the reinforcement group received feedback on every trial, strongly positive rewards (i.e., “Very Close”) were infrequent during early practice because errors were generally large, and when such rewards were received they may have been relatively unexpected. In contrast, the uncertainty associated with rewards in the supervision group quickly diminished with practice because errors became consistently small, and therefore large rewards were frequently obtained.

The poorer overnight retention exhibited by the supervision-based feedback group is consistent with the guidance hypothesis, which states that heavy guidance creates a dependency (Salmoni et al., 1984), and agrees with more recent work showing that vector-based error, i.e., that which indicates both error magnitude and the direction of decreasing error, elicits faster adaptation but worse retention compared to non-directional reward-based error feedback (Shmuelof et al., 2012). Others have speculated that heavy guidance prevents processing of other information important for the task (Winstein and Schmidt, 1990), which may impair learning.

### Transfer of a Novel Gait Pattern

In rehabilitation it is critical that what is learned on a treadmill transfers to an over-ground context. It was hypothesized that there would be superior transfer in the reinforcement-based feedback group. The rationale was that increased reward uncertainty in early practice would promote information seeking through exploration (Inglis et al., 1997; Anselme, 2010), exposing the learners to different movement patterns. Studies using upper extremity tasks have shown that more variable practice facilitates transfer (Kerr and Booth, 1978; Wrisberg and Liu, 1991; Green et al., 1995; Sherwood, 1996). However, the results did not support the hypothesis: both reinforcement and supervised groups had similar amount of transfer immediately after practice, and also overnight.

An unexpected result was that even though the supervised group had poor overnight retention, their overnight transfer did not suffer. The supervised group may have performed well on the immediate transfer and retention tests because any internal model learned during practice had relatively little time to decay at that point. However, model-based learning is associated with fast forgetting (Shmuelof et al., 2012), and therefore by the next-day retention test, their internal model may have decayed significantly. This begs the question: why was the supervised group able to perform well on the overnight transfer test, but performed poorly on the overnight retention test? This differential response may be due to differing contexts. When asked to perform in the same context as the initial learning (on a treadmill) in the overnight retention test, the supervised group may have tried to rely on a diminished internal model. However, when tested over-ground, the change in context may have prompted the retrieval of a different motor memory, one that is more resistant to forgetting. Instead of recalling a treadmill-based internal model, the participants may have remembered the actions that were rewarded during practice. The reinforcement group, on the other hand, may have not relied on an internal model to the same extent, and therefore performed well on both the overnight retention and transfer tests. This scenario assumes that multiple learning mechanisms were involved during task practice, one that is internal model-based and a model-free memory of successful actions (Huang et al., 2011).

### Reinforcement Learning and Task Redundancy

In the pilot experiment (Experiment 1) subjects were able to achieve the desired gait pattern by movements other than pure eversion due to cross-talk in the electrogonimeter measurements. This may be responsible for the substantially worse performance of the reinforcement group relative to the supervised group in Experiment 1. The task redundancy may have created a credit-assignment problem (Sutton, 1984), i.e., with relatively sparse categorical feedback, subjects might have had difficulty attributing a given result/reward with a particular action. This may have been less of an issue for the supervised group, who were guided directly to the desired ankle angle through instruction and feedback. In Experiment 2, the motion capture-based measurement system removed redundancy in the task goal—subjects could only achieve the desired gait pattern by eversion. Consequently, both the reinforcement and supervised groups performed similarly during the initial practice period.

### Implications for Gait Rehabilitation

Existing gait rehabilitation programs are mainly based on supervised learning principles—clinicians provide guidance to patients about how to alter their movements to achieve a desired gait pattern. This feedback can be either visual or haptic, but in either case, it usually provides information about both the degree of error and also directional information about how to reduce error. For example, clinicians may place visual cues on the floor, which shows the patient how to modify their gait to increase their step length (Morris et al., 1996; Suteerawattananon et al., 2004; Amatachaya et al., 2013), or may use a metronome that provides timing information, which patients can use to adjust their cadence (Howe et al., 2003; Suteerawattananon et al., 2004). In body-weight-supported treadmill training, therapists often provide manual assistance to move patients’ legs (Hesse et al., 1995). The results of the present study suggest that clinicians could benefit by shifting their role from being a supervisor that guides a patient into a desired gait pattern, to a reinforcer who rewards patients for achieving a desired gait pattern. The difference is subtle, but could be important for learning. Allowing patients to find healthier movement patterns on their own (health permitting), with direction in the form of rewards instead of guidance, may be a more rewarding learning experience that in turn improves long-term retention.

### Limitations and Considerations

While the data support the hypothesis that a reinforcement-learning based gait training approach improves retention of a new gait pattern, there are several points that should be considered before drawing broad conclusions from the results of this study. The training was of limited duration, and the retention/transfer tests only followed participants for one day. The trained gait pattern represents an unusual gait not observed in healthy adults. However, this was by design, as it provided a significant learning opportunity for subjects. It is unknown whether the results would be similar if the subjects were asked to learn a different gait pattern. During the transfer tests, subjects were asked to walk at the same speed as on the treadmill, but they could have deviated from the treadmill speed as their exact walking speed over-ground was not rigidly controlled. Finally, it is unknown whether the results would be similar for patient populations, who may have different gait abnormalities and cortical organizations, such as patients with chronic stroke.

## Conclusions

The results showed that subjects who received categorical error information in a reinforcement learning paradigm improved their task performance, retained what they learned, and transferred their learned gait pattern to an over-ground walking context. Moreover, participants who trained using this approach had better overnight retention than those who received directionally-specific error information in a supervised learning approach. The self-guided nature of reinforcement-based gait training may have provided a more rewarding experience for the participants, improving retention of a novel gait pattern.

## Funding

This research was supported by a Northeastern University TIER 1 Award (Internal Grant Program in Support of Interdisciplinary Research).

## Conflict of Interest Statement

The authors declare that the research was conducted in the absence of any commercial or financial relationships that could be construed as a potential conflict of interest.
